# Effects of recruitment manoeuvre on perioperative pulmonary complications in patients undergoing robotic assisted radical prostatectomy: A randomised single-blinded trial

**DOI:** 10.1371/journal.pone.0183311

**Published:** 2017-09-06

**Authors:** Eun-Su Choi, Ah-Young Oh, Chi-Bum In, Jung-Hee Ryu, Young-Tae Jeon, Hyoung-Gyun Kim

**Affiliations:** 1 Department of Anesthesiology and Pain Medicine, Nowon Eulji Medical Center, Eulji University, Seoul, Republic of Korea; 2 Department of Anesthesiology and Pain Medicine, Seoul National University Bundang Hospital, Seongnam, Republic of Korea; 3 Department of Anesthesiology and Pain Medicine, Konyang University Hospital, Daejeon, Republic of Korea; Eberhard Karls University, GERMANY

## Abstract

Robotic-assisted laparoscopic radical prostatectomy (RARP) needs a steep Trendelenburg position and a relatively high CO_2_ insufflation pressure, and patients undergoing RARP are usually elderly. These factors make intraoperative ventilatory care difficult and increase the risk of perioperative pulmonary complications. The aim was to determine the efficacy of recruitment manoeuvre (RM) on perioperative pulmonary complications in elderly patients undergoing RARP. A total of 60 elderly patients scheduled for elective RARP were randomly allocated to two groups after induction of anaesthesia; positive end expiratory pressure (PEEP) was applied during the operation without RM in the control group (group C) and after RM in the recruitment group (group R). The total number of patients who developed intraoperative desaturation or postoperative atelectasis was significantly higher in group C compared to group R (43.3% *vs*. 17.8%, *P* = 0.034). Intraoperative respiratory mechanics, perioperative blood gas analysis, and pulmonary function testing did not show differences between the groups. Adding RM to PEEP compared to PEEP alone significantly reduced perioperative pulmonary complications in elderly patients undergoing RARP.

## Introduction

Robotic-assisted laparoscopic radical prostatectomy (RARP) has attracted increasing attention because it has lower rates of complications and improves the surgical outcome compared to open radical prostatectomy [1, 2]. In terms of anaesthetic management, RARP reduces blood loss, lowers the rate of transfusion, and shortens the hospitalisation period in comparison to conventional prostatectomy [3].

To facilitate RARP, the operative position should be a Trendelenburg head-down position as much as possible. Therefore, the operative position for RARP has a 30° slope, which is much steeper than that of other surgeries. In addition, a relatively higher CO_2_ gas insufflation pressure, of up to 17 mmHg, is used to improve visualisation. The steep Trendelenburg head-down position and relatively long duration of CO_2_ pneumoperitoneum (generally more than 3 hours) can result in an increased risk of intraoperative hypoxia and postoperative atelectasis [4, 5]. In addition, an increase of PaCO_2_ can be difficult to control. Furthermore, patients undergoing RARP are mostly elderly and the likelihood of difficulty of management of intraoperative oxygenation, and the risk of postoperative pulmonary complications, are increased.

Atelectasis develops after the induction of general anaesthesia due to mechanical ventilation in 90% of patients [6]. To prevent postoperative atelectasis and to improve oxygenation, positive end expiratory pressure (PEEP), maintenance of muscle tone, recruitment manoeuvre (RM), and minimisation of absorption of gas can be used [7]. RM is an important component of lung-protective ventilation, which has proven to be beneficial in the ventilation of patients with acutely diseased lungs, such as those with acute respiratory distress syndrome or asthma [8]. However, the role of lung-protective ventilation, including RM, in the intraoperative setting is still not clear and needs to be elucidated [9, 10].

This study was designed to evaluate the efficacy of RM in addition to PEEP on intraoperative oxygenation, ventilatory mechanics, and perioperative pulmonary complications in patients undergoing RARP.

## Methods

This prospective single-blind randomised controlled study was approved by the Seoul National University Bundang Hospital Institutional Review Board (protocol number B-1306/206-004) and was registered at ClinicalTrials.gov (NCT02013011). After obtaining written informed consent, we enrolled patients aged 60–80 years with American Society of Anaesthesiology physical status 1 or 2, who were scheduled for RARP under general anaesthesia from November 4^th^ 2013 to December 29^th^ 2014. Exclusion criteria were as follows: overweight (BMI > 31 kg/m^2^), existing myocardial infarction, a history of cardiac disease, having a moderate or severe obstructive or restrictive pattern on pulmonary function testing, active pulmonary disease and heavy smoking, neuromuscular disease, having neurologic sequelae due to neurologic disease, dementia, and renal disease. A total of 60 eligible patients were randomly allocated to two groups using a computer-generated list.

Patients received midazolam 0.03 mg/kg as a premedication in the reception area of the operating theatre. After arrival at the operating room, patients were monitored with standard monitoring, including electrocardiography, non-invasive arterial pressure, and pulse oximetry. Anaesthesia was induced in all patients with propofol and remifentanil administered via target-controlled infusions (TCI) using an Orchestra infusion pump system (Fresenius Vial, Brezins, France) and a bolus of rocuronium. The concentrations of propofol and remifentanil were adjusted with TCI to maintain a bispectral index (BIS) of 40–60 (measured with an A-2000 BISTM monitor; Aspect Medical Systems Inc., Natick, MA, USA) and the mean arterial pressure and heart rate within 20% of pre-induction values during the maintenance of anaesthesia. The temperature was checked via skin temperature probe and was maintained at over 35°C. The arterial catheter was inserted at the radial artery to monitor blood pressure and arterial blood gas analysis.

After induction, 5 cmH_2_O of PEEP was applied to all patients without RM in the control group (group C) and after RM in the recruitment group (group R). We performed RM by using a ventilator as the alveolar recruitment method, following Whalen at al [11]. During RM, a tidal volume of 6–8 mL/kg of predicted body weight, ventilatory rate of 10 breaths/min, F_I_O_2_ of 0.4, and inspiratory:expiratory ratio of 1:2 in pressure control mode were maintained. Lungs were recruited by increasing the PEEP gradually, from 4 cmH_2_O (2 breaths) to 6 cmH_2_O (2 breaths), 8 cmH_2_O (2 breaths), and finally 16 cmH_2_O (10 breaths). After 10 breaths with 16 cmH_2_O, PEEP was decreased stepwise as before. We planned the recruitment method so that the peak airway pressure at the final point during RM did not exceed 35 cmH_2_O because patients were elderly and at risk for haemodynamic instability. After RM, the tidal volume of 6–8 mL/kg of predicted body weight was unchanged and 5 cmH_2_O PEEP was applied. Mechanical ventilation was started with the pressure control ventilation mode. The lungs were mechanically ventilated with an F_I_O_2_ of 0.4, inspiratory:expiratory ratio of 1:2, tidal volume of 6–8 mL kg^-1^ of predicted body weight, and ventilatory rate of 10 breaths/min. Ventilatory rate and inspiration pressure were adjusted to maintain end-tidal carbon dioxide tension of 4–6 kPa. All of these procedures were performed in the supine position. The application of PEEP was continued until the end of surgery. Patients whose saturation decreased below 95%, or exceeded 7.3 kPa of PaCO_2_, were excluded from this study and were managed accordingly irrespective of the study protocol. We recorded the number of these patients.

The patient-tested baseline pulmonary function testing was performed using a portable spirometer (MicroLoop^™^; Carefusion, Basingstoke, UK) on a day before operation, before leaving the post-anaesthetic care unit (PACU), and on the postoperative second day. Low-dose chest computed tomography (CT) was also performed on the postoperative second day for evaluation of postoperative atelectasis. During the intraoperative and PACU periods, arterial blood gas analyses were performed as baseline measurements before application of RM and PEEP (T1), 30 min after induction of CO_2_ pneumoperitoneum (T2), 90 min after induction of CO_2_ pneumoperitoneum (T3), and 30 min after arrival in the PACU in room air (T4).

We calculated gas exchange parameters using data from arterial blood gas analysis. The static compliance was calculated from the following formula: static compliance = expiratory tidal volume / (plateau inspiration pressure−PEEP). The dynamic compliance was calculated from the following formula: dynamic compliance = expiratory tidal volume / (peak inspiration pressure − PEEP) [12]. Alveolar oxygen pressure (PAO_2_) was obtained from the alveolar gas equation: PAO_2_ = (760–47) × F_I_O_2_—PaCO_2_ / 0.8. Alveolar–arterial gradient (AaDO_2_) was the difference between alveolar and arterial PO_2_.

The primary outcome was the incidence of perioperative pulmonary complications. Sample size calculation was based on a previous study in which the incidence of atelectasis after laparoscopic surgery was 30% [13]. We considered a reduction of the incidence by 90% to be statistically significant. Thus, we calculated that 30 patients would be needed in each group, using a two-sided test with 80% power and a two-sided 5% α-error, allowing for 20% dropouts. Data are expressed as means ± SD (standard deviation) or as number of patients. Statistical differences in nominal data were analysed by the chi-square test. Statistical differences in continuous data were compared using the t-test. SPSS for Windows software (ver. 20.0; SPSS Inc., Chicago, IL, USA) was used for statistical analyses. A *P* value < 0.05 was considered statistically significant.

## Results

**Fig 1:**
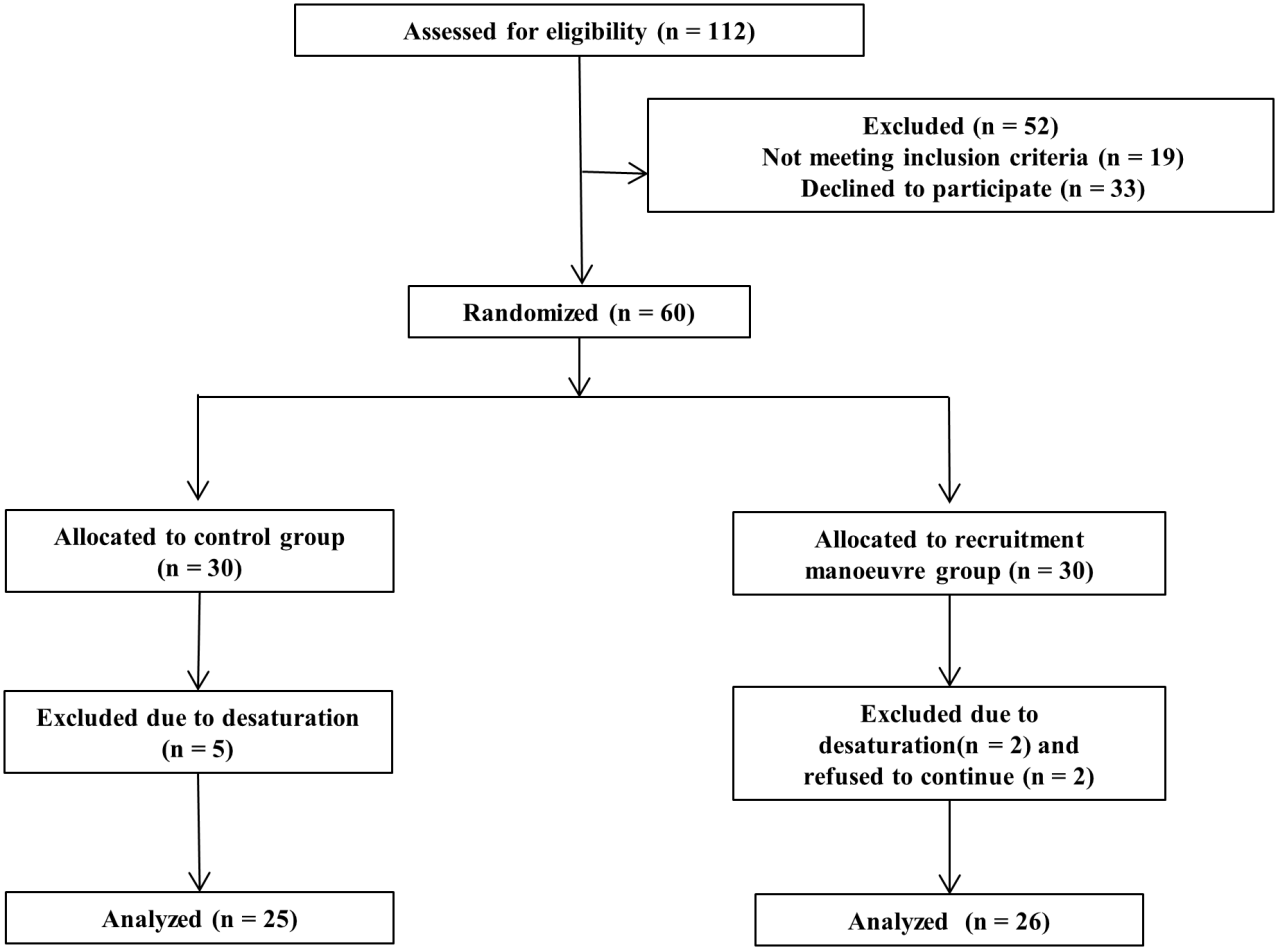
Flow chart of patient enrollment.

Of a total of 112 patients, 19 were excluded based on the exclusion criteria (Fig 1). Of the 93 patients who were screened as eligible, 33 declined to participate, and 60 were enrolled in the study and were randomly assigned to group C (n = 30) or group R (n = 30) (Table 1). There were no differences between the groups in terms of patient characteristics or operative data. The number of patients who dropped out due to decreased saturation during surgery was five in group C and two in group R. Two patients in group R was dropped out due to refusal to check Low-dose CT.

**Table 1:** Patient characteristics and operative data.

	Group C	Group R
	(n = 25)	(n = 26)
**Age (year)**	66.6 ± 4.3	67.6 ± 4.3
**Height (cm)**	169.0 ± 6.6	166.2 ±5.6
**Weight (kg)**	70.0 ± 7.1	68.7 ±7.1
**BMI (kg m**^**-2**^**)**	24.5 ± 2.1	24.5 ±2.0
**Operation time (min)**	205.8 ± 42.0	194.8 ± 30.1
**Anaesthesia time (min)**	255.0 ± 52.8	250.2 ± 30.5
**Estimated blood loss (mL)**	196.2 ± 130.3	150.4 ± 76.7
**Crystalloid (mL)**	1327.0 ± 338.9	1344.0 ± 384.1

Values are mean ± SD.

BMI, body mass index.

After induction, there were no differences in respiratory mechanical parameters or gas exchange parameters between the two groups (Table 2). Thirty minutes after formation of pneumoperitoneum, respiratory rate, peak inspiration pressure, and plateau inspiration pressure were significantly increased in both groups (P < 0.05) (Table 2). In addition, static compliance and dynamic compliance were significantly decreased in both groups (P < 0.05) (Table 2). Mean values of alveolar dead space ventilation to tidal volume ratio, PaO_2_, arterial/alveolar O_2_ tension (a/A ratio), AaDO_2_, and the ratio of partial pressure of arterial oxygen and fraction of inspired oxygen (PaO_2_/F_I_O_2_) showed a tendency to decline after pneumoperitoneum in both groups. In addition, PAO_2_ was significantly decreased after pneumoperitoneum in both groups (P < 0.01) (Table 3). Thirty minutes after pneumoperitoneum induction, there were no significant differences in respiratory mechanical parameters and gas exchange parameters between the two groups. Ninety minutes after pneumoperitoneum induction, both respiratory mechanical parameters and gas exchange parameters were similar between the groups.

**Table 2:** Respiratory mechanical parameters at each phase during surgery.

	Group C	Group R	*P -*value
	(n = 25)	(n = 26)	
**Tidal volume**			
**After induction**	416.1 ± 43.6	415.5 ± 41.0	0.50
**30 min after pneumoperitoneum**	400.7 ± 38.8	398.1 ± 34.6	0.74
**90 min after pneumoperitoneum**	408.6 ± 26.3	402.3 ± 39.4	0.64
**RR**			
**After induction**	10.2 ± 1.5	10.2 ± 1.4	0.86
**30 min after pneumoperitoneum**	12.5 ± 1.7	12.3 ± 2.2	0.65
**90 min after pneumoperitoneum**	13.2 ± 2.4	12.9 ± 2.6	0.71
**P peak**			
**After induction**	12.4 ± 2.5	13.1 ± 3.9	0.84
**30 min after pneumoperitoneum**	28.3 ± 3.2	28.3 ± 4.1	0.99
**90 min after pneumoperitoneum**	28.2 ± 3.0	28.3 ±3.6	0.94
**PIP**			
**After induction**	12.4 ± 2.6	12.9 ± 3.1	0.79
**30 min after pneumoperitoneum**	23.2 ± 3.0	23.1 ± 3.9	0.87
**90 min after pneumoperitoneum**	23.2 ± 2.9	22.1 ± 5.6	0.68
**Static compliance**			
**After induction**	34.8 ± 7.2	33.72 ± 8.6	0.60
**30 min after pneumoperitoneum**	22.5 ± 3.9	23.15± 9.7	0.60
**90 min after pneumoperitoneum**	23.3 ± 4.8	24.00 ± 7.4	0.88
**Dynamic compliance**			
**After induction**	34.8 ± 7.2	33.6 ± 8.9	0.64
**30 min after pneumoperitoneum**	17.5 ± 2.6	17.8 ± 4.9	0.68
**90 min after pneumoperitoneum**	17.8 ± 2.8	17.7 ± 3.0	0.59

Values are mean ± SD or numbers.

RR, respiratory rate.

P peak, plateau inspiration pressure.

PIP, peak inspiratory pressure.

**Table 3:** Gas exchange parameters at each phase during surgery.

	Group C	Group R	*P -*value
	(n = 25)	(n = 26)	
**ETCO**_**2**_			
**After induction**	33.7 ± 2.8	32.8 ± 2.7	0.60
**30 min after pneumoperitoneum**	35.1 ± 3.2	33.7 ± 3.5	0.10
**90 min after pneumoperitoneum**	35.4 ± 2.8	35.6 ± 2.8	0.88
**PCaO**_**2**_			
**After induction**	33.2 ± 2.7	33.3 ± 3.9	0.96
**30 min after pneumoperitoneum**	37.6 ± 3.0	37.2 ± 3.4	0.68
**90 min after pneumoperitoneum**	39.3 ± 3.8	39.6 ± 4.3	0.92
**PaO**_**2**_			
**After induction**	143.0 ± 44.9	152.7 ± 61.5	0.83
**30 min after pneumoperitoneum**	136.4 ± 39.3	143.7 ± 39.8	0.51
**90 min after pneumoperitoneum**	143.1 ±34.9	149.4 ± 36.8	0.85
**PAO**_**2**_			
**After induction**	243.7 ± 3.4	243.6 ± 4.7	0.96
**30 min after pneumoperitoneum**	238.2 ± 3.8	238.7 ± 4.3	0.77
**90 min after pneumoperitoneum**	236.1 ± 4.7	235.8 ± 5.4	0.92
**a/A ratio**			
**After induction**	0.6 ±0.2	0.6 ± 0.2	0.96
**30 min after pneumoperitoneum**	0.6 ± 0.2	0.6 ± 0.2	0.52
**90 min after pneumoperitoneum**	0.6 ± 0.2	0.6 ± 0.2	0.76
**AaDO**_**2**_			
**After induction**	100.7 ± 44.1	101.4 ± 40.6	1.0
**30 min after pneumoperitoneum**	101.9 ± 40.7	95.0 ± 39.8	0.55
**90 min after pneumoperitoneum**	93.0± 34.9	86.3 ± 35.4	0.74
**PaO**_**2**_**/F**_**I**_**O**_**2**_			
**After induction**	357.5 ± 112.4	381.8 ± 153.7	0.92
**30 min after pneumoperitoneum**	340.9 ± 98.3	359.4 ± 99.6	0.55
**90 min after pneumoperitoneum**	314.5 ± 90.9	311.9 ± 95.5	0.81

Values are mean ± SD or numbers.

ETCO_2_, end tidal carbon dioxide tension.

PaCO_2_, arterial carbon dioxide tension.

PaO_2_, arterial oxygen tension.

PAO_2,_ alveolar oxygen pressure.

AaDO_2_, arterial/alveolar O_2_ tension.

a/A, alveolar–arterial gradient.

PaO_2_/F_I_O_2_, ratio of partial pressure arterial oxygen and fraction of inspired oxygen.

In the PACU, there was no significant difference in gas exchange parameters between the two groups (Table 4). The results of pulmonary function testing were similar between the groups (Table 5). The number of patients who developed postoperative atelectasis was eight in group C and three in group R. The total number of patients who either developed postoperative atelectasis or dropped out due to decreased saturation during surgery was significantly higher in group C (P = 0.034) (Table 6). All patients who had an atelectasis on low-dose chest CT in group R did not have clinical symptoms. However, one patient who had an atelectasis on low-dose chest CT in group C developed pneumonia.

**Table 4:** Gas exchange parameters at PACU.

	Group C	Group R	*P -*value
	(n = 25)	(n = 26)	
**PaCO**_**2**_	34.0 ± 3.8	32.6 ± 3.5	0.20
**PaO**_**2**_	81.1 ±8.7	83.1 ± 11.7	0.49
**SO**_**2**_	95.7 ± 2.1	96.2 ± 2.5	0.61
**PAO**_**2**_	107.3 ± 4.7	109.0 ± 4.4	0.20
**a/A ratio**	0.8 ± 0.1	0.8 ± 0.1	0.68
**AaDO**_**2**_	26.2 ± 9.6	25.9 ± 12.4	0.88
**PaO**_**2**_**/F**_**I**_**O**_**2**_	386.0 ± 41.6	395.7 ± 56.0	0.49

Values are mean ± SD or numbers.

ETCO_2_, end tidal carbon dioxide tension.

PaCO_2_, arterial carbon dioxide tension.

PaO_2_, arterial oxygen tension.

SO_2_, peripheral capillary oxygen saturation.

PAO_2_, alveolar oxygen pressure.

AaDO_2_, arterial /alveolar O_2_ tension.

a/A, alveolar–arterial gradient.

PaO_2_/F_I_O_2_, ratio of partial pressure arterial oxygen and fraction of inspired oxygen.

**Table 5:** Results of pulmonary function testing.

	Group C	Group R	*P -*value
	(n = 25)	(n = 26)	
**FEV1**			
** Before surgery**	2.7 ± 1.5	2.4 ± 0.9	0.32
** In the PACU**	01.0 ± 0.9	1.0 ± 0.9	0.98
** POD 1 day**	2.0 ±0.6	1.7 ±1.1	0.59
**FVC**			
** Before surgery**	3.3 ± 2.1	3.0 ±0.9	0.92
** In the PACU**	1.3 ± 1.2	1.5 ± 1.2	0.68
** POD 1 day**	2.4 ± 0.8	2.1 ±1.2	0.65
**FEV1/FVC**			
** Before surgery**	77.6 ± 26.1	78.1 ± 21.6	0.68
** In the PACU**	48.7 ± 10.8	43.5 ± 38.9	0.84
** POD 1 day**	87.9 ± 10.8	77.2 ± 15.1	0.25

Values are mean ± SD or numbers.

PACU, post anaesthetic care unit.

FEV1, forced expiratory volume in 1 s.

FVC, forced vital capacity.

**Table 6:** Percent of patients who had complications.

	Group C	Group R	*P -*value
	(n = 25)	(n = 26)	
**Postoperative atelectasis**	8/25	3/26	0.08
**Decreased saturation**	5/30	2/28	0.24
**Perioperative pulmonary complication**	13/30	5/28*	0.03

Values are proportions.

* < 0.05 vs. control group.

## Discussion

This study showed that, compared with only PEEP, RM with PEEP can prevent postoperative pulmonary complications. In addition, this study indicated the potential of RM with PEEP as a method to manage intraoperative oxygenation.

Atelectasis easily develops after general anaesthesia due to mechanical ventilation [6]. In one report, the incidence of postoperative atelectasis was 100% in patients undergoing general anaesthesia, when they were tested using CT [14]. Causes of atelectasis are dyskinesis resulting from neuromuscular agents, high F_I_O_2_, and absence of the sigh breath [15]. Neuromuscular blocker-induced dyskinesis limits the movement of the dependent diaphragm, which leads to a decrease of lung compliance. Also, the movement of the dependent portion of the lung is limited and the functional residual capacity is decreased. High F_I_O_2_ causes absorption atelectasis. The sigh breath is a normal reflex that functions to maintain pulmonary compliance, minimise the alveolar-arterial oxygenation difference, and maintain venous admixture within the normal range. The absence of the sigh breath causes decreases of PaO_2_ and pulmonary compliance, which leads to atelectasis.

In RALP, the frequency of atelectasis can be increased according to the position of surgery and older age of the patients. For the best view of the operating field, RARP needs a maximally steep Trendelenburg position of more than 30°. Therefore, intraperitoneal organs compress the diaphragm and lungs. In addition, intraperitoneal pressure for pneumoperitoneum is about 17 mmHg, which is higher than the pressure used for other laparoscopic surgeries. Furthermore, most patients undergoing RALP are elderly. Because of the lower compliance of elderly patients compared to younger patients, and the age-related change in pulmonary function, the incidence of postoperative atelectasis is higher than in younger patients [6].

The RM is amplified as vital capacity breaths and a kind of sigh breath [16]. It is an artificial support to recruit collapsed alveoli and improve arterial oxygenation by increasing airway pressure, while sigh breaths are a physiologic reflex in awake patients. There are two RM methods. One method is to maintain inflation of the lungs for 5–30 seconds at a fixed peak inspiratory pressure [16–20]. The other method is to gradually increase PEEP in a stepwise manner [11, 21]. Both methods were reported to be equally effective [22]. We chose the second method because our patients were elderly and high, fixed peak inspiratory pressure leads to haemodynamic instability more easily than in younger patients.

In our study, after undergoing Trendelenburg positioning and formation of pneumoperitoneum, patients experienced significant increases in respiratory rate, peak inspiration pressure, and plateau inspiration pressure, and significant decreases in static and dynamic compliance. In addition, patients experienced a general decrease of gas exchange parameters. These results are similar to those of previous studies and represent predictable consequences [23–25]. However, the mean value of PaO_2,_ and the a/A ratio, of group R were higher than those of group C, but the differences were not statistically significant. In a previous study, the RM showed statistically significantly effectiveness in a similar number of patients [26]. In another study that compared PEEP and RM with PEEP, RM with PEEP maximised the effects of ventilation, including oxygenation, in open general surgery with general anaesthesia [27].

The cause of our results, in which the effect of RM was not as dramatic as in the previous study, was that the enrolled patients were elderly. In elderly patients, there is a reduction in the elastic recoil of the lungs [28], a condition characterised by a reduction in the alveolar surface area without alveolar destruction, associated with hyperinflation and reduced alveolar-capillary diffusing capacity. In other words, it is similar to emphysema. Thus, the portion of the lung re-expanded by the RM may re-collapse faster and more easily than in younger patients such that the RM is less effective. Furthermore, the pressure of pneumoperitoneum for RARP is higher than that for laparoscopic gynaecologic surgery. Therefore, the risk of atelectasis is higher and the effect of RM is attenuated. For these reasons, even though a previous study demonstrated that RM significantly improved arterial oxygenation for at least 30 min during laparoscopic surgery, RM in our study was effective, but not significantly so [26]. Furthermore, for these reasons, RM did not affect the postoperative spirometer results, and the results of pulmonary function testing were similar between the groups in our study. This finding is similar to a study that showed that RM did not affect postoperative pulmonary function in obese patients undergoing laparoscopic surgery [29].

In terms of perioperative pulmonary complications, although the difference was not statistically significant, the number of patients who dropped out during the intraoperative period was greater in group C and dropouts occurred within 30 minutes from the formation of pneumoperitoneum. From this result, we inferred that RM is helpful to maintain oxygenation and prevent atelectasis resulting from high pressure pneumoperitoneum. Also, the incidence of postoperative atelectasis was higher in group C, although the difference was not statistically significant. Furthermore, the incidence of dropout and postoperative atelectasis was statistically higher in group R than in group C. Therefore, RM can reduce pulmonary complications and may be helpful to reduce subclinical atelectasis and prevent respiratory complications. Our study has significance because it is the first to investigate the occurrence of atelectasis in RARP using a computer.

To effectively apply and lengthen the RM in RARP, additional studies with other methods are needed. First, repeated RM will be helpful to lengthen its effect as in a previous study of abdominal surgery [30]. In previous studies, a single implementation of RM was sufficiently effective to show statistical differences [11, 16, 21, 27, 31–33]. However, RARP is processed in the restricted and non-elastic pelvic space, and patients age is older compared to other types of operation. Therefore, repeated RM will more likely be needed to produce an effect. Another method is to apply much higher PEEP to lengthen the effect. Lee et al. evaluated the optimal PEEP for RARP and found that 7 cm H_2_O of PEEP is suitable for RARP [34]. In the case of obese patients undergoing general anaesthesia, 10 cm H_2_O of PEEP improved respiratory parameters and oxygenation [35, 36]. The other method is to change the inspiration:expiration ratio, to 1:1, for example [37]. A ratio of 1:1 can lower peak inspiration pressure and improve lung compliance [37, 38].

The main limitation of this study was the small number of enrolled patients. The reason for this was that our primary endpoint was incidence of perioperative pulmonary complications, and calculations were based on a previous study in which the subjects were younger than our patients, and which involved laparoscopic surgery rather than robotic surgery [13]. If more patients were enrolled in our study, we could have more clearly shown the effectiveness of RMs in elderly patients undergoing RARP.

## Conclusions

RM with PEEP reduced perioperative pulmonary complications in elderly patients undergoing RARP. Further studies are required to examine the effect of RM on pulmonary mechanics and gas-exchange parameters in this setting.

## Supporting information

S1 File CONSORT checklist. (DOC)

S2 File Clinical sudy protocol in English. (RTF)
