# Supplementary material for: Effects of recruitment manoeuvre on perioperative pulmonary complications in patients undergoing robotic assisted radical prostatectomy: A randomised single-blinded trial
Source: PLoS One. 2017 Sep 6;12(9):e0183311. doi: 10.1371/journal.pone.0183311 (PMC5587235; doi:10.1371/journal.pone.0183311)
Supplement: S2 File — (RTF) [file pone.0183311.s002.rtf]

Research proposal for IRB approval


Effects of recruitment maneuver in patients undergoing robotic assisted radical prostatectomy	


2013. 7.

Seoul National University Bundang Hospital
 Department of Anesthesiology & Pain medicine

Ah-Young Oh


< Summary of proposal >

Title	Effects of recruitment maneuver in patients undergoing robotic assisted radical prostatectomy	
Background	- There is increasing attention for robotic assisted radical prostatectomy (RARP) for lower rates of complications and better surgical outcome.
- In RARP, the operative position should be a Trendelenburg head-down position of 30¡Æslope, which is much steeper than that of other surgeries. 
-The steep Trendelenburg head-down position and relatively long duration of CO2 pneumoperitoneum can result in an increased risk of intraoperative hypoxia and postoperative atelectasis. Furthermore, patients undergoing RARP are mostly elderly and the risk of pulmonary complications is increased. 
- Recently, positive end expiratory pressure(PEEP) and recruitment maneuver(RM) are commonly used during general anesthesia to improve oxygenation. It is known that using PEEP during laparoscopic procedures is beneficial, on the contrary benefit of RM is not established, especially in RARP. 	
Purpose	This study is designed to evaluate the efficacy of RM on intraoperative oxygenation, ventilatory mechanics, and postoperative pulmonary complications.	
Method	- A total of 60 patients, aged 60-80 years with ASA class 1 or 2, scheduled for elective RARP under general anesthesia will be enrolled and randomly allocated to two groups.
- Positive end expiratory pressure (PEEP) will be applied 20 minutes after induction of anesthesia, without RM in the control group (group C) and after RM in the recruitment group (group R). 
- Induction and maintenance of anesthesia will be done with propofol and remifentanil using target-controlled infusions (TCI).
- During surgery, a tidal volume of 6–8 mL/kg of predicted body weight, PEEP of 5 cmH2O, FIO2 of 0.4, inspiratory:expiratory ratio of 1:2, PIP less than 35 cmH2O in pressure control mode will be maintained, and RR will be adjusted to maintain ETCO2 of 35~40 mmHg. 
- Arterial blood gas analysis is going to be performed as baseline measurements before application of RM and PEEP (T1), 30 min after induction of CO2 pneumoperitoneum (T2), 90 min after induction of CO2 pneumoperitoneum (T3), and 30 min after arrival in the PACU in room air (T4) to assess oxygenation status.
- Pulmonary function tests will be done preoperatively and postoperatively to assess changes in pulmonary function after surgery, and chest CT scan will be done postoperatively to check lung complications, such as atelectasis.
- Exclusion criteria are as follows: overweight (BMI > 31 kg/m2), existing myocardial infarction, a history of cardiac disease, having a moderate or severe obstructive or restrictive pattern on pulmonary function testing, active pulmonary disease and heavy smoking, neuromuscular disease, having neurologic sequelae due to neurologic disease, dementia, and renal disease. 	
Principal researcher	Ah-Young Oh, Department of Anesthesiology & Pain medicine	
Research period	1 year from IRB approval (assumed to be ~2014.8)	
Financial suppot	Research program of the Seoul National University Bundang Hospital	


Research proposal
1. Title
Effects of recruitment maneuver in patients undergoing robotic assisted radical prostatectomy

2. Institution
Seoul National University Bundang Hospital, Department of Anesthesiology & Pain medicine
82, Gumi-ro 173 Beon-gil, Bundang-gu, Seongnam-Si, Gyeonggi-Do 463-707, South Korea

3. Researchers
Ah-Young Oh, Department of Anesthesiology and Pain Medicine, Seoul National University Bundang Hospital
Eun-Su Choi, Department of Anesthesiology and Pain Medicine, Seoul National University Bundang Hospital
Chi-Bum In, Department of Anesthesiology and Pain Medicine, Chungnam National University Hospital
Jung-Hee Ryu, Department of Anesthesiology and Pain Medicine, Seoul National University Bundang Hospital
Young-Tae Jeon, Department of Anesthesiology and Pain Medicine, Seoul National University Bundang Hospital
Sun-Woo Nam, Department of Anesthesiology and Pain Medicine, Seoul National University Bundang Hospital
4. Financial support
Research Program of the Seoul National University Bundang Hospital

5. Background 
5.1 Increasing demand for Robotic assisted radical prostatectomy(RARP)
- Robotic-assisted laparoscopic radical prostatectomy (RARP) has attracted increasing attention because it has lower rates of complications and improves the surgical outcome compared to open radical prostatectomy. [1, 2]
- In terms of anaesthetic management, RARP reduces blood loss, lowers the rate of transfusion, and shortens the hospitalisation period in comparison to conventional prostatectomy. [3]

5.2 Characteristics of RARP
- To facilitate RARP, the operative position should be a Trendelenburg head-down position as much as possible. 
- Therefore, the operative position for RARP has a 30¡Æslope, which is much steeper than that of other surgeries. 
- In addition, a relatively higher CO2 gas insufflation pressure, of up to 17 mmHg, is used to improve visualisation. 
- The steep Trendelenburg head-down position and relatively long duration of CO2 pneumoperitoneum (generally more than 3 hours) can result in an increased risk of intraoperative hypoxia and postoperative atelectasis [4, 5]. In addition, an increase of PaCO2 can be difficult to control. 
- Furthermore, patients undergoing RARP are mostly elderly and the likelihood of difficulty of management of intraoperative oxygenation, and the risk of postoperative pulmonary complications, are increased. 

5.3 Measures for preventing atelectasis and improving oxygenation during general anesthesia
- Atelectasis develops after the induction of general anaesthesia due to mechanical ventilation in 90% of patients [6]. 
- To prevent postoperative atelectasis and to improve oxygenation, positive end expiratory pressure (PEEP), maintenance of muscle tone, recruitment manoeuvre (RM), and minimisation of absorption of gas can be used [7]. 

5.4 Application of PEEP and RM in RARP
- RM is an important component of lung-protective ventilation, which has proven to be beneficial in the ventilation of patients with acutely diseased lungs, such as those with acute respiratory distress syndrome or asthma [8]. 
- PEEP and RM both can be used in RARP to improve oxygenation.
- PEEP can be applied during surgery constantly.
- RM is expected to improve oxygenation during surgery, but whether RM is effective in RARP is yet to be proven. Especially due to steep trendelenburg head down position and relatively long duration of CO2 pneumoperitoneum, it is hard to predict how much effect RM will have or whether RM can influence postoperative lung care. 

5.5 Pre-existing studies
- In RARP, regarding ventilatory management there are only studies about the difference between volume controlled mode and pressure controlled mode or whether PEEP improves oxygenation during surgery. [9, 10]

5.6 Research objective
- This study was designed to evaluate the efficacy of RM in addition to PEEP on intraoperative oxygenation, ventilatory mechanics, and perioperative pulmonary complications in patients undergoing RARP. 

6. Subjects
   Patients undergoing elective RARP under general anesthesia

7. Research period
  1 year from IRB approval (assumed to be ~2014.8)

8. Recruiment of participants
8.1. Inclusion and exclusion criteria
Inclusion criteria
¨ç age 60-80 years 
¨è ASA class 1, 2 
¨é scheduled for RARP under general anesthesia
¨ê written informed consent

Exclusion criteria
¨ç history of myocardial infarction 
¨è valvular heart disease
¨é moderate or severe obstructive or restrictive pattern on pulmonary function testing
¨ê heavy smoker
¨ë neurologic sequelae due to neurologic disease
¨ì renal insufficiency
¨í overweight (BMI > 31 kg/m2)

Dropout criteria
Patient¡¯s refusal to participate 
Change in surgical plan to other procedure than RARP
Patients whose saturation decrease below 95%, or PaCO2 exceed 7.3 kPa 

8.2 Number of participants
     Control group(C group): 30
     Recruitment group(R group): 30

9. Methods
10.1. Enrollment
 The patients who are undergoing elective RARP under general anesthesia and adequate for inclusion & exclusion criteria will be enrolled after informed consent. 

10.2. Informed consent
Researchers will obtain written forms of consent after sufficient explanation about the research using preorganized explanatory note.

10.3. Ramdomisation
Before registration of participants, a block assign list according to the random number table will be made, which consists of blocks sized 4 which contains 2 patients from control group(A) and 2 patients from experimental group(B) (AABB, BBAA, ABAB, BABA, ABBA, BAAB), and blocks sized 6 which contains 3 patients from each group (AAABBB, AABABB, AABBAB, AABBBA, BBBAAA, BBABAA, BBAABA, BBAAAB, ABAABB, ABABAB, ABABBA, ABBBAA, ABBAAB, ABBABA, BAAABB, BAABAB, BAABBA, BABBAA, BABABA, BABAAB). The participants will be assigned to control group and experimental group according to this random assign list. 
All the process of randomisation will be conducted by an anesthesiologist who is not involved in the study, and the assignment will be kept in a sealed envelope which is not accessible to the researchers until induction of anesthesia. 

10.4. Sample size
  The primary outcome is the incidence of perioperative pulmonary complications. Sample size calculation was based on a previous study in which the incidence of atelectasis after laparoscopic surgery was 30% [11]. We considered a reduction of the incidence by 90% to be statistically significant. Thus, we calculated that 30 patients would be needed in each group, using a two-sided test with 80% power and a 5% ¥á-error, allowing for 20% dropouts. 

10.5. Study protocol
All the clinical procedures other than RM will be conducted in same measures in both group.
- The patients who are undergoing elective RARP under general anesthesia will be enrolled.
- The participants will be randomly allocated into 2 groups.
- After induction, 5 cmH2O of PEEP will be applied to all patients without RM in the control group (group C) and after RM in the recruitment group (group R). 
-	Participants will be premedicated with 2 mg of midazolam.
- Induction and maintenance of anesthesia will be done with propofol and remifentanil using an Orchestra infusion pump system (Fresenius Vial, Brezins, France). The concentrations of propofol and remifentanil wil be adjusted with TCI to maintain a bispectral index (BIS) of 40–60 (measured with an A-2000 BISTM monitor; Aspect Medical Systems Inc., Natick, MA, USA). 
- Muscle relaxation will be done before tracheal intubation using 0.6 mg/kg of rocuro nium.
- During surgery, a tidal volume of 6–8 mL/kg of predicted body weight, PEEP of 5 cmH2O, FIO2 of 0.4, inspiratory:expiratory ratio of 1:2, PIP less than 35 cmH2O in pressure control mode will be maintained, and RR will be adjusted to maintain ETCO2 of 35~40 mmHg. 
- Lungs are going to be recruited by increasing the PEEP gradually, from 4 cmH2O (2 breaths) to 6 cmH2O (2 breaths), 8 cmH2O (2 breaths), and finally 16 cmH2O (10 breaths). After 10 breaths with 16 cmH2O, PEEP is going to be decreased stepwise as before. 
- The surgical procedure will be conducted using robotic sytem(da Vinci; Intuitive Surgicla, Inc., Sunnyvale, CA), and intraabdominal pressure of 17 mmHg will be maintained during the procedure.
- During the intraoperative and PACU periods, arterial blood gas analyses will be performed as baseline measurements before application of RM and PEEP (T1), 30 min after induction of CO2 pneumoperitoneum (T2), 90 min after induction of CO2 pneumoperitoneum (T3), and 30 min after arrival in the PACU in room air (T4). 
- The patient-tested baseline pulmonary function testing will be performed using a portable spirometer (MicroLoop¢â; Carefusion, Basingstoke, UK) on a day before operation, before leaving the post-anaesthetic care unit (PACU), and on the postoperative second day. 


- Low-dose chest computed tomography (CT) will be performed on the postoperative second day for evaluation of postoperative atelectasis. 
10.6. Monitoring parameters and laboratory tests
1) Patient demographics and monitoring parameters
Patients¡¯age, height, weight, BMI, comorbidities, past medical histories, medications, operation time, anesthesia time, estimated blood loss, amounts of crystalloid and colloid infused during surgery, transfusion, respiratory index (tidal volume, RR, ETCO2, PIP, PEEP, AaDo2, static & dynamic compliance, FIO2,), blood pressure and heart rate changes before and after RM will be documented.

2) Laboratory and clinical tests
Arterial blood gas analysis (pH, pCO2, pO2, Lactate, hematocrit, HCO3-, TCO2, base excess, saturation, hemoglobin), alveolar-arterial oxygen tension difference, pre- and postoperative pulmonary function test, chest CT scan will be conducted.

10.7. Outcome variables and analyses
1) Primary outcome – the incidence of perioperative pulmonary complications
2) Secondary outcome – differences in static & dynamic compliance of each group, changes in pulmonary function test results
3) Other variables – differences in arterial blood gas analysis results, blood pressure and heart rate changes before and after RM 

10.8. Safety assessment
 Safety assessment will be conducted for all the patients who are included in the study. If an adverse event occurs, it will be documented with whether there is any causal relationship between the event and research methods, and its severity, importance, duration will be assessed afterwards. Interventions for adverse events and their results will also be documented. Adverse events will be assessed and intervened immediately according to any abnormal test results, and additional tests other than planned ones can be done if nessessary.

1)Severity
Adverse events will be classified into 3 classes according to following definitions:
Mild adverse event is temporary and does not cause impairment in daily activies.
Moderate adverse event causes slight impairment in daily activies.
Severe adverse event causes major impairment in daily activies.

2)Cause - result relationship
Cause - result relationship will be classified into 5 classes: ¡®definitely related', 'highly likely to be related', 'likely to be related', 'not likely to be related', 'difficult to determine causalty'.

3)Category of adverse events 
Adverse events related to PEEP or RM
Pneumothorax occurs rarely due to PEEP or RM, but it is known that small amount of pneumothorax tends to be spontaneously resolved. Also, because this adverse event generally occurs in process of applying PEEP or conducting RM, there is no difference expected in the category of adverse events between R group and C group.

10.9. Statistical analysis
SPSS for Windows software (ver. 19.0; SPSS Inc., Chicago, IL, USA) will be used for statistical analyses. Patients¡¯age, height, weight, BMI, comorbidities, past medical histories, medications, operation time, anesthesia time, estimated blood loss, amounts of crystalloid and colloid infused during surgery, transfusion, respiratory index (tidal volume, RR, ETCO2, PIP, PEEP, AaDo2, static & dynamic compliance, FIO2,) arterial blood gas analysis (pH, pCO2, pO2, Lactate, hematocrit, HCO3-, TCO2, base excess, saturation, hemoglobin), alveolar-arterial oxygen tension difference, pulmonary function test results will be analysed and compared between 2 groups. 
Statistical differences in nominal data will be analysed by the chi-square test. Statistical differences in continuous data will be compared using the Kruskal–Wallis test. Post-hoc subgroup analyses will be performed with the Mann–Whitney U test. A P value < 0.05 will be considered statistically significant. 

11. Reference
1) Hu JC, Gu X, Lipsitz SR, Barry MJ, D'Amico AV, Weinberg AC, Keating NL. Comparative effectiveness of minimally invasive vs open radical prostatectomy. JAMA 2009; 302: 1557-64.
2) Menon M, Shrivastava A, Tewari A. Laparoscopic radical prostatectomy: conventional and robotic. Urology 2005; 66: 101-4.
3) D'Alonzo RC, Gan TJ, Moul JW, Albala DM, Polascik TJ, Robertson CN, Sun L, Dahm P, Habib AS. A retrospective comparison of anesthetic management of robot-assisted laparoscopic radical prostatectomy versus radical retropubic prostatectomy. J Clin Anesth 2009; 21: 322-8. 
4) Safran DB, Orlando R. Physiologic effects of pneumoperitoneum. Am J Surg 1994; 167: 281—6.
5) Tokics   L,   Hedenstierna   G,   Strandberg   A,   Brismar   B, Lunquist H. Lung collapse and gas exchange during general anesthesia. effects of spontaneous breathing, muscle paralysis,  and  positive  end-expiratory pressure.  Anesthesiology 1987; 66: 157—67. 
6) Gunnarsson L, Tokics L, Gustavsson H, Hedenstierna G. Influence of age on atelectasis formation and gas exchange impairment during general anaesthesia. Br J Anaesth 1991; 66 : 423-32.
7) Hedenstierna G, Rothen HU. Atelectasis formation during anesthesia: causes and measures to prevent it. J Clin Monit Comput 2000; 16: 329-35.
8) Tusman G, Böhm SH, Suarez-Sipmann F, Turchetto E. Alveolar recruitment improves ventilatory efficiency of the lungs during anesthesia. Can J Anaesth. 2004; 51: 723-7.
9) Choi EM, Na S, Choi SH, An J, Rha KH, Oh YJ. Comparison of volume-controlled and pressure-controlled ventilation in steep Trendelenburg position for robot-assisted laparoscopic radical prostatectomy. J Clin Anesth 2011; 23: 183-8. 
10) Meininger D, Byhahn C, Mierdl S, Westphal K, Zwissler B. Positive end-expiratory pressure improves arterial oxygenation during prolonged pneumoperitoneum. Acta Anaesthesiol Scand 2005; 49: 778-83.
11) Karayiannakis AJ, Makri GG, Mantzioka A, Karousos D, Karatzas G. Postoperative pulmonary function after laparoscopic and open cholecystectomy. Br J Anaesth 1996; 77(4): 448–452
